# Supplementary material for: Hybrid Models and Biological Model Reduction with PyDSTool
Source: PLoS Comput Biol. 2012 Aug 9;8(8):e1002628. doi: 10.1371/journal.pcbi.1002628 (PMC3415397; doi:10.1371/journal.pcbi.1002628)
Supplement: Text S4 — Complete source code for the PyDSTool package (version 0.88.120504). Includes API documentation and help files linking to web pages. This file is identical to the current public release on Sourceforge.net. (ZIP) [file pcbi.1002628.s004.zip › PyDSTool/html/PyDSTool.PyCont.TestFunc-module.html]

xml version="1.0" encoding="ascii"?


PyDSTool.PyCont.TestFunc


| Home | Trees | Indices | Help | PyDSTool |
| --- | --- | --- | --- | --- |

Package PyDSTool ::
Package PyCont ::
Module TestFunc

[hide private]

[frames] | no frames]

# Module TestFunc

source code

Test functions

Drew LaMar, March 2006


|  |  |
| --- | --- |
| Classes | [hide private] |

Function  
F: R^n --> R^m

TestFunc  
You need to define the function yourself within an inherited class.

BorderMethod  
Border method:

BiAltMethod

AddTestFunction  
Only works with testfuncs that don't have PreTestFunc and rely only
on sysfunc.

DiscreteMap  
Turns a function into a map composed with itself period times.

FixedPointMap  
Turns a discrete map into a fixed point map.

B\_Check  
There is an attempt here to be a little efficient.

Branch\_Det

Branch\_Bor

Fold\_Det

Fold\_Tan

Fold\_Bor

Hopf\_Det

Hopf\_Bor

Hopf\_Eig

Hopf\_Double\_Bor\_One

Hopf\_Double\_Bor\_Two

BT\_Fold

CP\_Fold

DH\_Hopf

BT\_Hopf\_One

GH\_Hopf\_One

BT\_Hopf

GH\_Hopf

LPC\_Det

PD\_Det

NS\_Det

ParTestFunc

UserDefinedTestFunc


|  |  |
| --- | --- |
| Variables | [hide private] |

\_classes = `['Function', 'TestFunc', 'AddTestFunction', 'Discre...`


|  |  |
| --- | --- |
| Variables Details | [hide private] |

### \_classes

Value:
:   ```
    ['Function',
     'TestFunc',
     'AddTestFunction',
     'DiscreteMap',
     'FixedPointMap',
     'BiAltMethod',
     'BorderMethod',
     'B_Check',
    ...
    ```

  


| Home | Trees | Indices | Help | PyDSTool |
| --- | --- | --- | --- | --- |

|  |  |
| --- | --- |
| Generated by Epydoc 3.0.1 on Fri May 4 15:24:03 2012 | http://epydoc.sourceforge.net |
